# Cytotoxic effects of replication-competent adenoviruses on human esophageal carcinoma are enhanced by forced p53 expression

**DOI:** 10.1186/s12885-015-1482-8

**Published:** 2015-06-10

**Authors:** Shan Yang, Kiyoko Kawamura, Shinya Okamoto, Suguru Yamauchi, Masato Shingyoji, Ikuo Sekine, Hiroshi Kobayashi, Yuji Tada, Koichiro Tatsumi, Kenzo Hiroshima, Hideaki Shimada, Masatoshi Tagawa

**Affiliations:** 1 Division of Pathology and Cell Therapy, Chiba Cancer Center Research Institute, 666-2 Nitona, Chuo-ku, 260-8717 Chiba Japan; 2 Department of Molecular Biology and Oncology, Graduate School of Medicine, Chiba University, Chiba, Japan; 3 Department of Biochemistry, Graduate School of Pharmaceutical Sciences, Chiba University, Chiba, Japan; 4 Department of Respirology, Graduate School of Medicine, Chiba University, Chiba, Japan; 5 Division of Respirology, Chiba Cancer Center, Chiba, Japan; 6 Department of Pathology, Tokyo Women’s Medical University Yachiyo Medical Center, Chiba, Japan; 7 Department of Surgery, School of Medicine, Toho University, Tokyo, Japan

**Keywords:** Esophageal carcinoma, Replication-competent adenovirus, p53, Apoptosis

## Abstract

**Background:**

Improvement of transduction and augmentation of cytotoxicity are crucial for adenoviruses (Ad)-mediated gene therapy for cancer. Down-regulated expression of type 5 Ad (Ad5) receptors on human tumors hampered Ad-mediated transduction. Furthermore, a role of the p53 pathways in cytotoxicity mediated by replication-competent Ad remained uncharacterized.

**Methods:**

We constructed replication-competent Ad5 of which the E1 region genes were activated by a transcriptional regulatory region of the *midkine* or the *survivin* gene, which is expressed preferentially in human tumors. We also prepared replication-competent Ad5 which were regulated by the same region but had a fiber-knob region derived from serotype 35 (AdF35). We examined the cytotoxicity of these Ad and a possible combinatory use of the replication-competent AdF35 and Ad5 expressing the wild-type *p53* gene (Ad5/p53) in esophageal carcinoma cells. Expression levels of molecules involved in cell death, anti-tumor effects in vivo and production of viral progenies were also investigated.

**Results:**

Replication-competent AdF35 in general achieved greater cytotoxic effects to esophageal carcinoma cells than the corresponding replication-competent Ad5. Infection with the AdF35 induced cleavages of caspases and increased sub-G1 fractions, but did not activate the autophagy pathway. Transduction with Ad5/p53 in combination with the replication-competent AdF35 further enhanced the cytotoxicity in a synergistic manner. We also demonstrated the combinatory effects in an animal model. Transduction with Ad5/p53 however suppressed production of replication-competent AdF35 progenies, but the combination augmented Ad5/p53-mediated p53 expression levels and the downstream pathways.

**Conclusions:**

Combination of replication-competent AdF35 and Ad5/p53 achieved synergistic cytotoxicity due to enhanced p53-mediated apoptotic pathways.

**Electronic supplementary material:**

The online version of this article (doi:10.1186/s12885-015-1482-8) contains supplementary material, which is available to authorized users.

## Background

Esophageal squamous cell carcinoma, which frequently developed in aged persons, remains intractable despite a recent progress in multi-modal treatments. Adenoviruses (Ad)-mediated gene transfer is a possible strategy to improve the prognosis by transducing the tumor cells with an exogenous therapeutic gene. Type 5 Ad (Ad5), which utilize the coxsackie adenovirus receptor (CAR) molecules as one of the major cellular receptors, are commonly used as a gene transfer vector and Ad5-mediated transduction is subjected to an expression level of CAR on the target cells [1]. Expression of CAR, ubiquitously detected in normal epithelia, is often down-regulated in various types of human tumors including esophageal carcinoma, which results in inefficient Ad5-mediated transduction [2, 3]. In contrast, type 35 Ad (Ad35) use CD46 molecules as a main cellular receptor and expressed levels of CD46 are well maintained even in human tumors [4, 5]. Previous studies showed that chimeric Ad5 vectors of which the fiber-knob portion was replaced by that of Ad35 (AdF35) infected target cells with the same efficacy as Ad35 [1, 6]. The AdF35 can thereby be a vector to transduce low CAR-expressing cells which are resistant to Ad5 infection. The differential receptor usage has also an advantage in simultaneously transferring dual genes into the same target cells. Ad5 infection down-regulated CAR expression levels on target cells through receptor internalization and hampered subsequent Ad5-mediated transduction. AdF35-mediated transduction was however not impaired by precedent Ad5 infection since the down-regulated receptor expression was exclusive each other [7].

Replication-competent Ad, designed to propagate specifically in tumors followed by spread of the viral progenies into neighboring tumor cells, can overcome low transduction efficacy and subsequently augment anti-tumor effects [8]. We previously demonstrated that a transcriptional regulatory region of the *midkine* (*MK*) or the *survivin* (*Sur*) gene, expressed in a number of human tumors but scarcely in normal tissues [9, 10], effectively activated an exogenous gene preferentially in tumors [11–13]. Moreover, we demonstrated that replication-competent Ad5 of which the *E1* genes were activated by the MK regulatory region produced anti-tumor effects on hepatocellular carcinoma [14].

Ad5 expressing the wild-type *p53* gene (Ad5/p53) have been clinically in use for cancer treatments and produced combinatory anti-tumor effects with chemotherapeutic agents [15, 16]. We also demonstrated that Ad5/p53 produced cytotoxic effects on human esophageal carcinoma and that the cytotoxicity was linked with CAR expression levels [17]. These results raise a possibility that enhanced p53 expression in combination with replication-competent Ad augments the anti-tumor effects. In this study, we examined cytotoxicity of replication-competent AdF35 powered by regulatory region of MK (AdF35-MK) or Sur (AdF35-Sur) on a panel of human esophageal carcinoma cells and examined a possible combinatory effect of Ad5/p53 and the AdF35.

## Methods

### Cells and mice

Human esophageal squamous cell carcinoma lines, TE-1, TE-2, TE-10, TE-11, YES-2, YES-4, YES-5, YES-6 and T.Tn cells, from Cell Resource Center for Biomedical Research, Tohoku University, Sendai, Japan, were cultured with RPMI 1640 medium supplemented with 10 % fetal calf serum. The *p53* genotype of respective tumors is shown in Table 1. Human embryonic kidney (HEK) 293 cells and human lung carcinoma A549 cells, from American Type Culture Collection (Manassas, VA, USA), were cultured with DMEM medium supplemented with 10 % fetal calf serum. BALB/c nu/nu mice (5-6 week-old females) were purchased from Japan SLC (Hamamatsu, Japan).

**Table 1:** Infectivity of Ad5 and AdF35 to esophageal carcinoma cells and CAR expression levels

Cells	p53 status	Mutated codon	CAR expression	GFP-positive cells (%)	
				Ad5	AdF35
TE-1	mutated	Codon 272	6.57	8.06 ± 1.17	53.47 ± 0.10
TE-2	wild-type		19.22	0.79 ± 0.14	10.15 ± 0.44
TE-10	mutated	Codon 242	14.73	16.15 ± 0.52	35.33 ± 0.67
TE-11	wild-type		22.25	22.86 ± 0.53	42.82 ± 0.74
YES-2	mutated	Codon 236	0.19	5.09 ± 1.29	51.54 ± 0.36
YES-4	wild-type		26.87	27.18 ± 0.16	61.23 ± 0.07
YES-5	mutated	Codon 280	15.40	22.18 ± 0.32	69.97 ± 0.89
YES-6	wild-type		30.23	16.59 ± 0.25	27.63 ± 0.17
T.Tn	mutated	Codon 214 and 258	8.12	0.49 ± 1.00	21.60 ± 0.15
HEK293			56.97	87.20 ± 0.52	78.05 ± 0.70

Cells were infected with Ad5/GFP or AdF35/GFP at a 30 MOI and were analyzed for the fluorescence with flow cytometry. Averages and the SEs are shown (*n* = 3). CAR expression levels were determined with flow cytometry and are expressed with an arbitrary unit

### Ad preparation

Replication-incompetent Ad5 DNA bearing the wild-type *p53*, the *green fluorescent protein* (Ad5/GFP) and the *β-galactosidase* gene (Ad5/LacZ) were constructed by ligation of transgene-harboring pShuttle 2 (Takara, Tokyo, Japan) and Adeno-X vector (Takara). Ad35 DNA bearing the above transgenes (AdF35/GFP, AdF35/LacZ) was produced with Adeno-X vector substituted with the Ad35 fiber-knob region. The fiber-knob modified Adeno-X DNA was created by replacing a fragment containing the Ad35 fiber-knob region (Avior therapeutics, Seattle, WA) (AY271307 at 30,827–33,609) with that of Ad5-derived region. The replication-incompetent Ad used the cytomegalovirus promoter to activate the transgene. Replication-competent Ad DNA of which the *E1* genes were activated by a transcriptional regulatory region of the *MK* or the *Sur* gene (Ad5/MK, AdF35/MK, Ad5/Sur, AdF35/Sur) were prepared with the regulatory sequences-harboring pShuttle 2 and Adeno-X vector or the fiber-knob replaced Adeno-X vector. The Ad DNA was transfected into HEK293 cells and the Ad were purified with an Adeno-X purification kit (Takara).

### Infectivity of Ad and receptor expression

Cells were infected with Ad5/GFP or AdF35/GFP at 30 multiplicity of infection (MOI) for 30 min and were washed to remove the Ad. They were cultured for 2 days and were analyzed for the fluorescence with FACSCalibur and CellQuest software (BD Biosciences, San Jose, CA, USA). Cell populations that showed fluorescence greater than the brightest 5 % of uninfected cells were judged as positively stained. Cells were stained with anti-CAR antibody (Ab) (Upstate, Charlottesville, VA, USA) followed by fluorescein isothiocyanate-conjugated anti-mouse IgG Ab, and were analyzed for their fluorescence intensity with FACSCalibur and CellQuest software. The mean fluorescence intensity of the stained cells was expressed as an arbitrary FL1 unit.

### Cell cycle analysis

Cells were fixed in 100 % ethanol, treated with RNase (50 μg/ml) and stained with propidium iodide (50 μg/ml). Cell cycle distributions were analyzed with FACSCalibur and CellQuest software.

### In vitro cytotoxicity assay and cell proliferation

Cells were seeded in 96-well plates (5x10^3^/well), infected with Ad at different amounts of virus particles (vp) and were cultured for 5–6 days. Cell viability was determined with a cell-counting WST kit (Wako, Osaka, Japan). The amount of formazan produced in each well was determined with the absorbance at 450 nm and the relative viability was calculated based on the absorbance without any treatments. Combinatory effects between AdF35/MK or AdF35/Sur and Ad5/p53 were examined with CalcuSyn software (Biosoft, Cambridge, UK). A combination index (CI) value was calculated based on the cell viability test with various vp of AdF35/MK or AdF35/Sur and Ad5/p53 (1.25 × 10^4^ vp/cell), and were shown at respective fractions affected (Fa) points. A CI value equal to 1 denotes an additive interaction, above 1 antagonism and below 1 synergism. Live cell numbers were calculated with a trypan blue dye exclusion test.

### Western blot analysis

Cell lysate was subjected to sodium dodecyl sulfate polyacrylamide gel electrophoresis. The protein was transferred to a nylon filter and was hybridized with Ab against Ad hexon, 14-3-3σ, glyceraldehyde-3-phosphate dehydrogenase (GAPDH) (Abcam, Cambrige, UK), Ad E1A (Santa Cruz Biotech, Santa Cruz, CA, USA), pRb, phosphorylated pRb, poly (ADP-ribose) polymerase (PARP) (that detected cleaved PARP), caspase-3, cleaved caspase-3, caspase-8 (that detected cleaved caspase-8), caspase-9 (that detected cleaved caspase-9), Fas-associated protein with death domain (FADD), death receptor 5 (DR5), Bcl-2, Bcl-xL, Bid (that detected truncated Bid (t-Bid)), phosphorylated p53 at Ser 15 or at Ser 46, Atg5, LC3A/B, Beclin-1, Bax, Puma, Fas, p21 (Cell Signaling, Danvers, MA, USA), p53 (Lab Vision, Fremont, CA, USA) or α-tubulin (Thermo Fisher Scientific, Fremont, CA, USA). The membranes were developed with the ECL system (GE Healthcare, Buckinghamshire, UK).

### Animal experiments

YES-2 cells (1 × 10^6^) were subcutaneously injected into BALB/c *nu/nu* mice and the mice received an intratumoral injection of Ad (2.5 × 10^8^ plaque-forming unit (pfu)/mouse) 4 times. In combinatory experiments, mice received intratumoral injection of AdF35 (1.875 × 10^8^ pfu/mouse) and/or Ad5 (1.875 × 10^8^ pfu/mouse) when the tumors reached to about 65 mm^3^ in volume. Tumor volumes were calculated according to the formula (1/2 × length × width^2^). The animal experiments with human cell lines were approved by the animal experiment and welfare committee at Chiba University (Reference number H26-242, H26-244). They were performed according to the guideline on animal experiments and were in compliance with the Helsinki Declaration.

### Virus production

YES-2 cell lysate after Ad infection was examined for the cytotoxicity with A549 cells and the virus titers were calculated with the median tissue culture infectious dose (TCID_50_) method.

## Results

### Improved transduction efficacy with AdF35

We compared infectivity of Ad5 and AdF35 vectors to 9 kinds of human esophageal carcinoma cells using GFP-expressing Ad with flow cytometry (Table 1). The differential fluorescence intensity of Ad5/GFP and AdF35/GFP in the same cells was influenced by the Ad infectivity since the same cytomegalovirus promoter was used to activate the *GFP* gene. The esophageal carcinoma cells were relatively resistant to Ad5-mediated transduction compared with HEK293 cells. All the esophageal cells showed greater infectivity with AdF35/GFP than with Ad5/GFP in contrast to HEK293 cells which showed similar transduction with Ad5/GFP and AdF35/GFP.

### Compared cytotoxicity of replication-competent AdF35 and Ad5

**Fig. 1:**
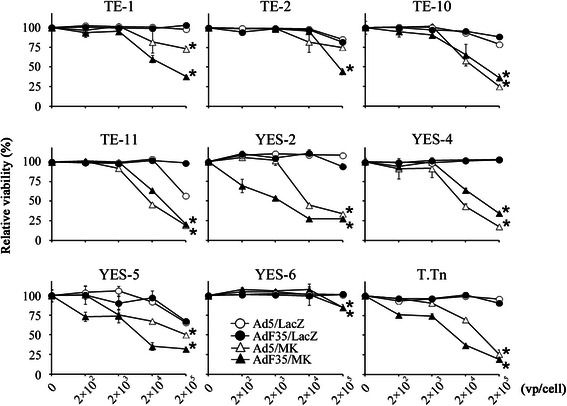
Enhanced cytotoxicity of AdF35/MK. Viability of esophageal carcinoma cells that were treated with various doses of Ad5/MK, AdF35/MK, Ad5/LacZ or AdF35/LacZ was examined with the WST assay. The relative viability was calculated based on the absorbance without any treatments. Standard errors (SE) bars are shown (*n* = 3). ^*^P < 0.01; comparing between Ad5/MK- or AdF35/MK-infected cells and Ad5/LacZ- or AdF35/LacZ-infected cells

We examined cytotoxic activities of replication-competent AdF35 and Ad5 on esophageal carcinoma cells, and compared the activities between Ad5/MK and AdF35/MK (Fig. 1), and Ad5/Sur and AdF35/Sur, with the same cells (Additional file 1: Figure S1). Susceptibility to replication-competent Ad was different among the cells tested, but AdF35 produced greater cytotoxicity than corresponding Ad5 in some of cells irrespective of the regulatory region. AdF35/MK achieved cytotoxic effects relatively greater than Ad5/MK in TE-1, YES-2, YES-5 and T.Tn cells, whereas the cytotoxicity between the 2 kinds of Ad was similar in other cells and YES-6 cells were resistant to the Ad-mediated cytotoxicity. AdF35/Sur also produced greater cytotoxicity than Ad5/Sur in TE-1, TE-2, TE-10, YES-2 and T.Tn cells, whereas both types of Ad showed similar cytotoxicity in other cells. AdF35/LacZ and Ad5/LacZ did not influence the viability or was minimally inhibitory at the high Ad doses. These data collectively suggest that AdF35 held an advantage in the cytotoxicity over Ad5 in some of the cells such as TE-1, YES-2 and T.Tn cells.

### Cell cycle changes induced by Ad infections

**Fig. 2:**
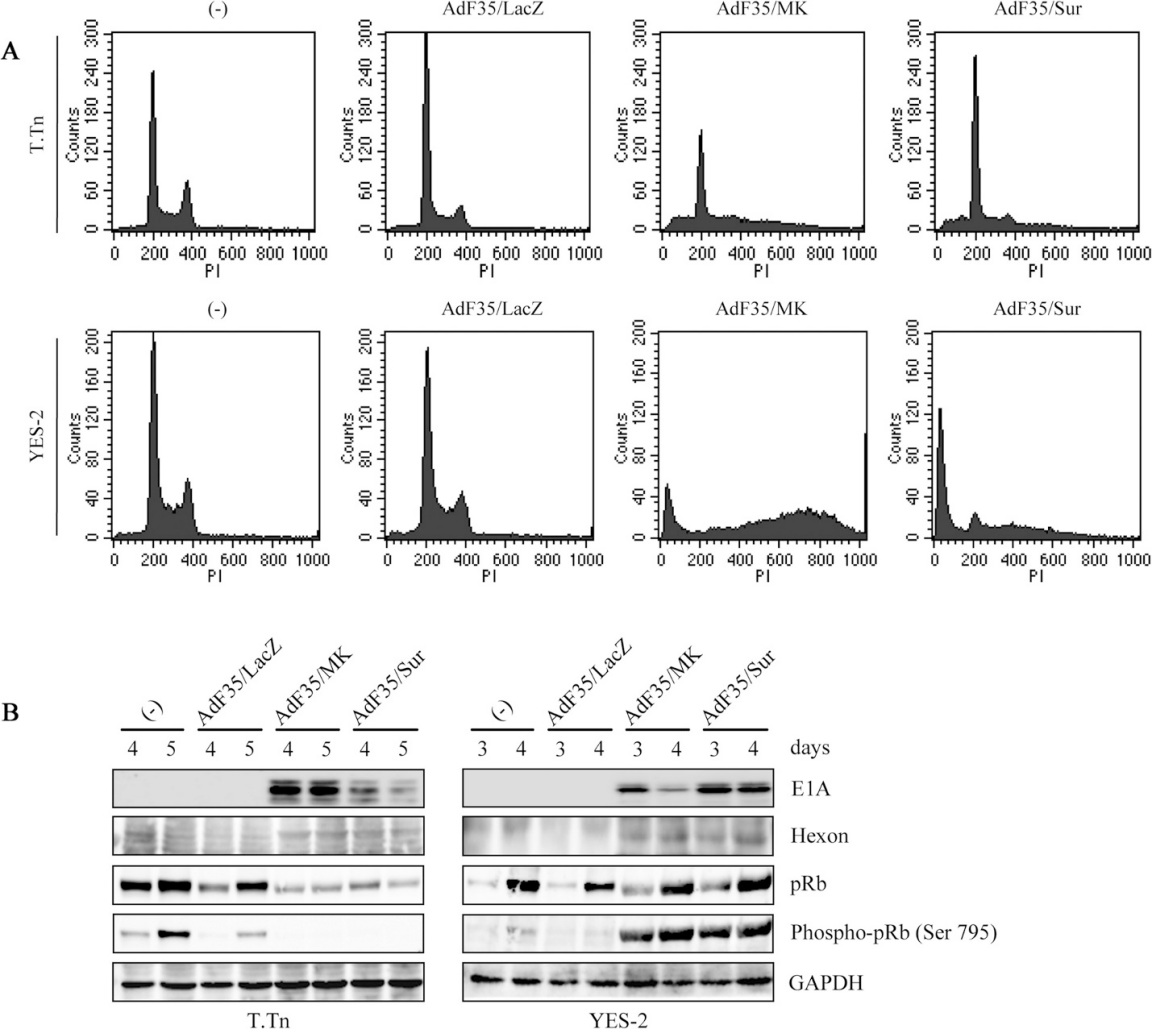
Cell cycle changes and expressed viral proteins of replication-competent Ad. T.Tn cells and YES-2 cells were uninfected or infected with AdF35/LacZ, AdF35/MK or AdF35/Sur (1 × 10^4^ vp/cell), and cultured for the indicated time. **a** Representative flow cytometrical analyses about cell cycle changes in YES-2 (day 3) and T.Tn cells (day 4). **b** Expression levels of viral proteins and pRb examined with western blot analyses. GAPDH was used as a loading control

We examined cell cycle distributions caused by AdF35 infections in T.Tn and YES-2 cells (Fig. 2a) (Additional file 2: Table S1). AdF35/MK- and AdF35/Sur-infected cells initially increased a fraction with more than 4 N populations (hyperploidy) and subsequently sub-G1 populations in a time dependent manner. AdF35/LacZ-infected cells did not show any significant changes in cell cycle compared with uninfected cells. Uninfected T.Tn and YES-2 cells showed a small percentage of hyperploidy fractions and AdF35/LacZ infection did not influence the hyperploid percentages.

### Replication-competent Ad activated apoptotic pathways

**Fig. 3:**
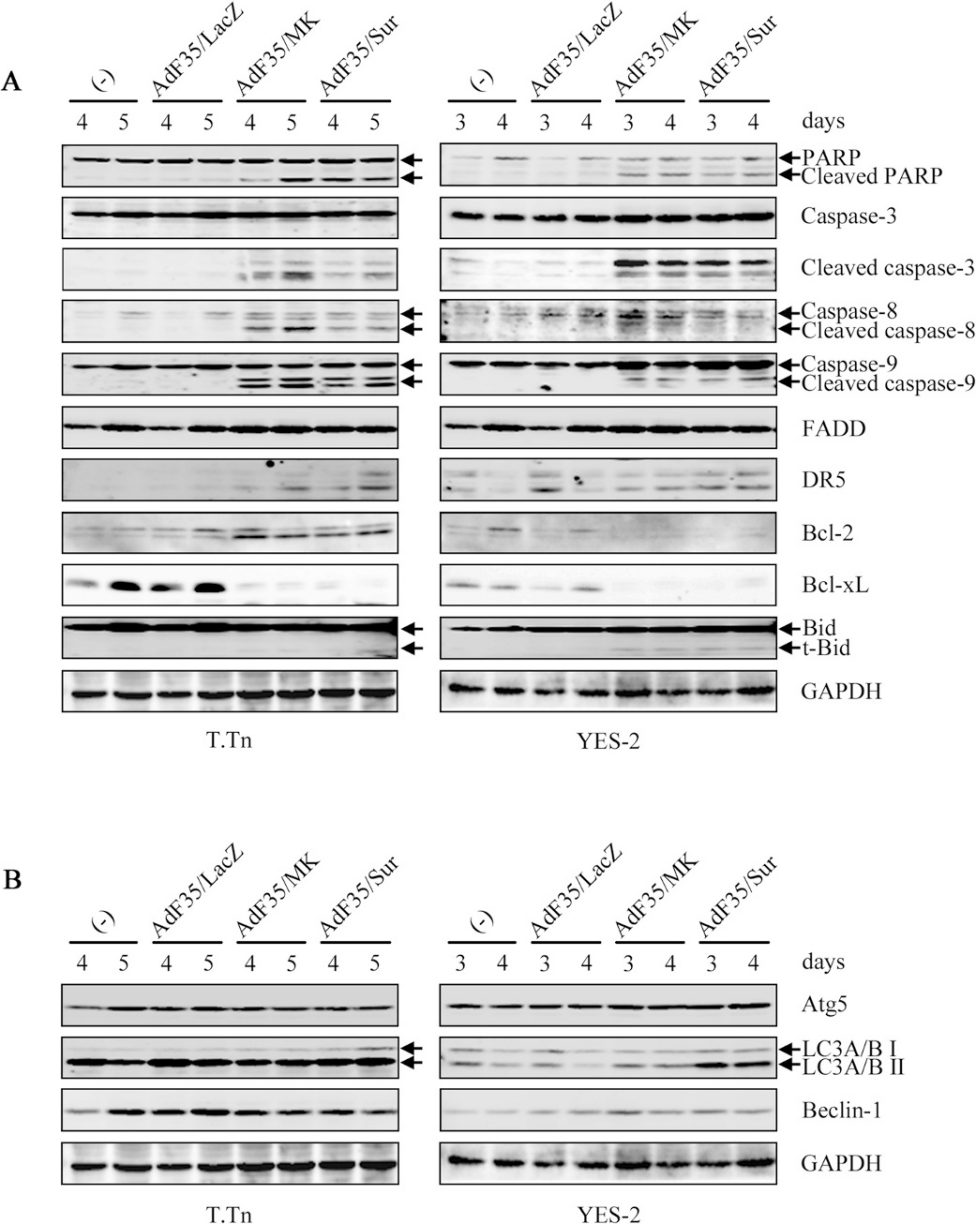
Replication-competent Ad-induced activation of apoptosis pathways. T.Tn cells and YES-2 cells were uninfected or infected with AdF35/LacZ, AdF35/MK or AdF35/Sur (1 × 10^4^ vp/cell), and cultured for the indicated time. **a** Expression levels of apoptosis- and **b** autophagy-linked proteins were examined with western blot analyses. GAPDH was used as a loading control

We further examined molecular events caused by replication-competent Ad with western blot analyses (Figs. 2b and 3). Transduction of T.Tn and YES-2 cells with AdF35/MK or AdF35/Sur but not AdF35/LacZ induced E1A, one of the early viral proteins, and hexon, the late viral protein (Fig. 2b). Interestingly, expression levels of pRb and phosphorylated pRb were down-regulated in T.Tn cells infected with AdF35/MK or AdF35/Sur, whereas those of phosphorylated pRb in the AdF35/MK- or AdF35/Sur-infected YES-2 cells were rather up-regulated. We then examined possible activation of apoptosis pathways (Fig. 3a). Transduction with AdF35/MK or AdF35/Sur but not AdF35/LacZ induced cleavage of PARP and caspase-3 in both cell lines, demonstrating replication-competent Ad achieved apoptotic cell death. Ad-infected T.Tn cells showed cleavage of both caspase-8, involved in the extrinsic cell death, and caspase-9, involved in the intrinsic mitochondria-mediated cell death, whereas Ad-infected YES-2 cells showed cleavage of caspase-9 but not casepase-8. Minimal changes of t-Bid were detected only in YES-2 cells. We also examined expression levels of molecules in an upstream pathway of the extrinsic apoptosis and found that expression levels of FADD and DR5, one of the tumor necrosis factor-related apoptosis-inducing ligand receptors, remained unchanged in YES-2 cells, but DR5 slightly increased in T.Tn cells. Moreover, we found that Bcl-2 and Bcl-xL expression was down-regulated in Ad-infected YES-2 cells, whereas up-regulated Bcl-2 with down-regulated Bcl-xL was detected in Ad-infected T.Tn cells. The differential regulations between Bcl-2 and Bck-xL could be due to ability to hold a balance between apoptosis and anti-apoptosis in T.Tn cells. These data thereby demonstrated that both the intrinsic and the extrinsic pathways were activated in T.Tn cells, whereas the intrinsic apoptosis was preferentially induced in YES-2 cells. We further investigated expression levels of Atg5, Beclin-1, and LC3A/B I and II, which were linked with the autophagy pathway (Fig. 3b). These levels were scarcely influenced by AdF35/MK or AdF35/Sur except slight increase of LC3A/B II in YES-2 cells. These data suggested that AdF35/MK and AdF35/Sur induced caspase-mediated apoptotic pathways with a cell type-dependent involvement of the extrinsic pathway, and that autophagy pathway minimally contributed to the Ad-mediated cytotoxicity.

### Replication-competent Ad produced anti-tumor effects in vivo

We investigated the anti-tumor effects of replication-competent Ad in an animal model (Additional file 3: Figure S2). Nude mice bearing subcutaneous YES-2 tumors were treated with intratumoral injections of AdF35/MK, AdF35/Sur or AdF35/LacZ as a control. Administration of AdF35/MK and AdF35/Sur but not AdF35/LacZ retarded the subsequent tumor growth.

### Combinatory effects of replication-competent AdF35 and Ad5/p53

**Fig. 4:**
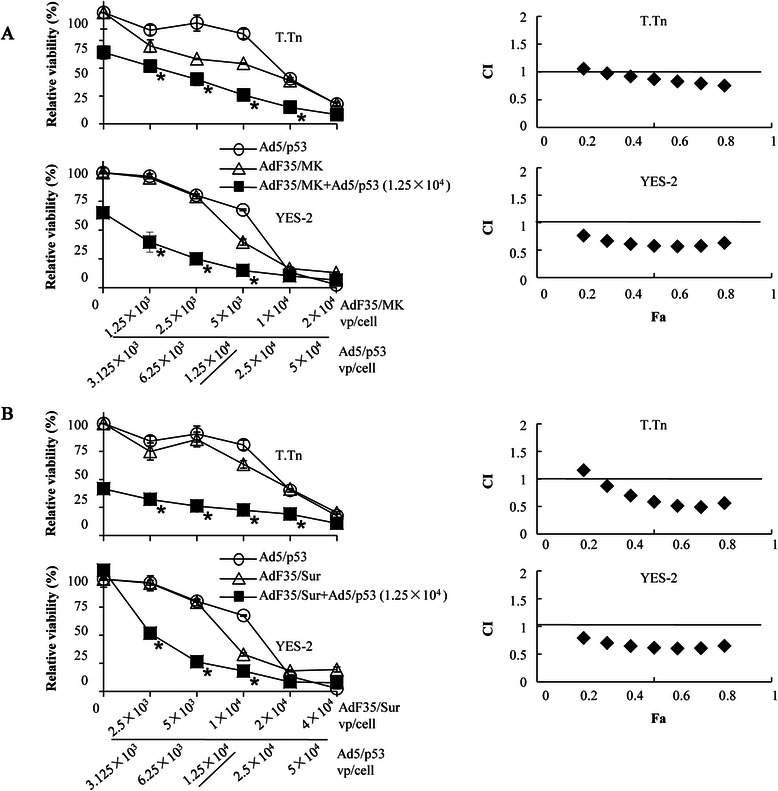
Combinatory cytotoxicity of replication-competent Ad and Ad5/p53. **a** Cells were infected with AdF35/MK, Ad5/p53, or AdF35/MK (at indicated doses) plus Ad5/p53 (1.25x10^4^ vp/cell). Relative viability of cells was examined with the WST assay and SE bars are shown (*n* = 3). CI values in the combination of AdF35/MK and Ad5/p53 in respective Fa points were shown. **b** Cells were infected with AdF35/Sur, Ad5/p53, or AdF35/Sur plus Ad5/p53 (1.25x10^4^ vp/cell). Relative viability of cells was examined with the WST assay and SE bars are shown (*n* = 3). CI values in the combination of AdF35/Sur and Ad5/p53 in respective Fa points were shown. CI < 1, CI = 1 and CI > 1 indicate synergistic, additive and antagonistic actions, respectively. ^*^P < 0.01; comparing between (A) AdF35/MK + Ad5/p53- or (B) AdF35/Sur + Ad5/p53-infected cells and (A) AdF35/MK-, Ad5/p53-, (B) AdF35/Sur- or Ad5/p53-infected cells

We examined possible combinatory effects between AdF35/MK or AdF35/Sur and Ad5/p53 (Fig. 4). Both YES-2 and T.Tn cells had mutated *p53* genes and were susceptible to Ad5/p53-mediated cytotoxicity [17]. These cells were infected with different amounts of replication-competent AdF35 or Ad5/p53, or in combination with AdF35 and Ad5/p53. The combinatory treatments with AdF35/MK and Ad5/p53 produced greater cytotoxicity than individual Ad alone in both cells (Fig. 4a). The CI values at Fa points between 0.3 and 0.8 in T.Tn cells or 0.2 and 0.8 in YES-2 cells indicated the combination produced synergistic effects. Transduction with Ad5/p53 also enhanced AdF35/Sur-induced anti-tumor effects in both cells and the CI values at the same Fa ranges showed synergism in the cytotoxicity between AdF35/Sur and Ad5/p53 (Fig. 4b).

### Growth inhibition by replication-competent AdF35 and Ad5/p53

**Fig. 5:**
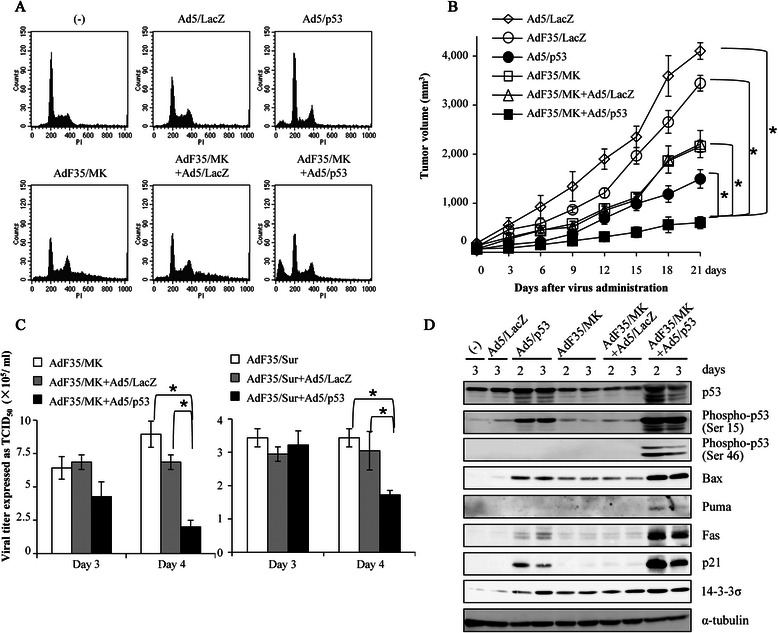
Production of anti-tumor effects with replication-competent Ad and Ad5/p53. **a** Representative flow cytometrical analyses about cell cycle changes. YES-2 cells were uninfected or infected with AdF35/MK (1.2 × 10^3^ vp/cell), Ad5/p53, Ad5/LacZ (6.5 × 10^3^ vp/cell) or in combination, and were analyzed on day 2. **b** Anti-tumor effects *in vivo* produced by AdF35/MK and Ad5/p53. Nude mice that were subcutaneously injected with YES-2 cells (1x10^6^) were treated with Ad as indicated (1.875x10^8^ pfu/mouse) on day 0, 3, 6 and 9 after the tumors developed to 65 mm^3^ (*n* = 5). The tumor sizes were measured every 4 days. Means and SE bars are shown. ^*^P < 0.01. **c** Influence of Ad/p53 on viral proliferation of replication-competent AdF35. YES-2 cells were infected with AdF35/MK or AdF35/Sur, or in combination of AdF35/MK or AdF35/Sur and Ad5/p53 or Ad5/LacZ (AdF35/MK and AdF35/Sur: 1.2 × 10^3^ vp/cell; Ad5/p53 and Ad5/LacZ: 6.5 × 10^3^ vp/cell), and the cell lysate was extracted on day 3 or 4. The viral titers were assayed with TCID_50_ method with A549 cells. Means and SE bars are shown (*n* = 3). ^*^P < 0.05. **d** Activation of the Ad5/p53-derived p53 pathways with AdF35/MK. YES-2 cells were uninfected or infected with AdF35/MK (1.2 × 10^3^ vp/cell), Ad5/p53 or Ad5/LacZ (6.5 × 10^3^ vp/cell) as a control, or in combination of AdF35/MK and Ad5/p53 or Ad5/LacZ for 2 or 3 days. Expression levels of molecules in the p53 pathways were analyzed with western blot analyses and those of α-tubulin were used as a loading control

We also investigated combinatory effects of replication-competent AdF35 and Ad5/p53 on cell proliferation (Table 2). YES-2 cells were infected with replication-competent AdF35 and with Ad5/p53 or Ad5/LacZ, and then cell numbers were counted. A combinatory use of AdF35/MK or Ad5F35/Sur and Ad5/p53 inhibited cell proliferation greater than AdF35/MK, AdF35/Sur or Ad5/p53 alone. We then examined cell cycle changes after the combinatory treatments with AdF35/MK and Ad5/p53 (Fig. 5a) (Additional file 4: Table S2). Infection with Ad5/p53 slightly increased G0/G1 and sub-G1 fractions in YES-2 cells, and AdF35/MK infection resulted in induction of hyperploidy populations. In contrast, transduction with both AdF35/MK and Ad5/p53 rather decreased hyperploidy fractions and greatly increased sub-G1 populations, suggesting that the combination augmented apoptotic cell death.

**Table 2:** Combinatory effects of AdF35/MK or AdF35/Sur and Ad5/p53 on cell growth

Treatment	Time (day)	Cell numbers (×10^4^)
(-)	2	174.3 ± 4.4
Ad5/LacZ	2	185.9 ± 2.4
Ad5/p53	2	98.3 ± 4.1^b^
AdF35/MK	2	133.7 ± 6.1^a^
AdF35/MK + Ad5/LacZ	2	141.9 ± 7.1
AdF35/MK + Ad5/p53	2	28.0 ± 2.1^d^
AdF35/Sur	2	147.7 ± 1.2^a^
AdF35/Sur + Ad5/LacZ	2	146.3 ± 1.8
AdF35/Sur + Ad5/p53	2	24.3 ± 3.0^d^
(-)	4	747.3 ± 5.1
Ad5/LacZ	4	718.2 ± 10.0
Ad5/p53	4	57.7 ± 5.6^b^
AdF35/MK	4	67.7 ± 7.0^b^
AdF35/MK + Ad5/LacZ	4	72.2 ± 1.5
AdF35/MK + Ad5/p53	4	3.3 ± 0.3^d^
AdF35/Sur	4	160.0 ± 3.0^b^
AdF35/Sur + Ad5/LacZ	4	134.9 ± 1.2
AdF35/Sur + Ad5/p53	4	19.3 ± 2.3^d^
(-)	6	1717.7 ± 1.3
Ad5/LacZ	6	1838.6 ± 7.2
Ad5/p53	6	1.2 ± 0.4^b^
AdF35/MK	6	12.0 ± 0.6^b^
AdF35/MK + Ad5/LacZ	6	20.3 ± 0.3
AdF35/MK + Ad5/p53	6	0 ± 0^c^
AdF35/Sur	6	40.3 ± 1.3^b^
AdF35/Sur + Ad5/LacZ	6	87.0 ± 3.8
AdF35/Sur + Ad5/p53	6	0 ± 0^c^

YES-2 cells were uninfected or infected with Ad as indicated (AdF35/MK and AdF35/Sur: 1.2× 10^3^ vp/cell; Ad5/p53 and Ad5/LacZ: 6.5× 10^3^ vp/cell). Live cell numbers were determined with a trypan blue dye exclusion test. Mean percentages with SEs are shown (*n* = 3)

^a^*P* < 0. 05, ^b^*P* < 0. 01; comparing between Ad/p53-, AdF35/MK- or AdF35/Sur-infected cells, and uninfected or Ad5/LacZ-infected cells. ^c^*P* < 0. 05, ^d^*P* < 0. 01; comparing between AdF35/MK + Ad5/p53- or AdF35/Sur + Ad5/p53-infected cells, and AdF35/MK-, AdF35/Sur-, Ad5/p53-, AdF35/MK + Ad5/LacZ-infected or AdF35/Sur + Ad5/LacZ-infected cells

### In vivo anti-tumor effects by AdF35/MK and Ad5/p53

We investigated the anti-tumor effects produced by Ad5F35/MK and Ad5/p53 in an animal model (Fig. 5b). Nude mice injected with YES-2 cells were treated with intratumoral administrations of Ad5F35/MK and/or Ad5/p53. Tumor growth of mice treated with AdF35/MK and Ad5/p53 was retarded compared with that of mice injected with either AdF35/MK or Ad5/p53 alone. In contrast, Ad5/LacZ treatments did not produce any combinatory effects with AdF35/MK although AdF35/LacZ alone slightly inhibited the tumor growth compared with Ad5/LacZ injections.

### Mechanisms of combinatory effects

We examined whether the enhanced anti-tumor effects of replication-competent AdF35 by Ad5/p53 infection were associated with increased production of the viral progenies. YES-2 cells were infected with AdF35/MK or AF35/Sur and Ad5/p53, and the cell lysate was tested for the viral amounts produced (Fig. 5c). Ad5/p53 infection significantly suppressed propagation of AdF35/MK or AdF35/Sur. The enhanced apoptosis by co-infected Ad5/p53 was not thereby due to increased production of infectious Ad progenies, but could be linked with augmented p53 functions. We then examined p53 activation processes with western blot analyses (Fig. 5d). Transduction of YES-2 cells with Ad5/p53 increased p53 expression levels and the phosphorylation at Ser 15 residues, whereas AdF35/MK infection did not augment the p53 and the phosphorylation. In contrast, a combinatory use of AdF35/MK and Ad5/p53 up-regulated p53 phosphorylation both at Ser 15 and Ser 46 residues, activation markers of the p53 pathways. Ad5/LacZ did not modulate endogenous p53 expression levels or induce the phosphorylation. We further examined the p53 downstream pathways. Ad/p53 infection induced expression of Bax, Fas, p21 and 14-3-3σ, and AdF35/MK infection stimulated expression of Bax and 14-3-3σ. The combination of both Ad further augmented expression levels of Bax, Fas, p21 and 14-3-3σ, and induced Puma expression. These data showed that AdF35/MK activated Ad5/p53-mediated p53 pathways.

## Discussion

In this study, we demonstrated that replication-competent AdF35 regulated by an exogenous transcriptional regulatory region induced apoptotic cell death in esophageal carcinoma and that combination of the AdF35 and Ad5/p53 produced synergistic cytotoxicity through enhanced activation of the p53 pathways. A direct injection of 2 kinds of Ad vectors into esophageal carcinoma is technically feasible and the up-regulated p53 pathways by the combinatory Ad usage can also contribute to enhanced sensitivity to chemotherapeutic agents and radiotherapy.

Fiber-modified AdF35 targeting CD46 molecules have advantages. One is high infectivity of AdF35 to human tumors since CD46 expression levels are maintained in tumors in contrast to CAR which is often down-regulated in the expression [18]. Another is to transduce cells which are infected with Ad5 vectors and consequently to enable simultaneous dual gene transfer. We took an advantage of the mutually uninhibited infection with AdF35 and Ad5 vectors and examined possible combinatory cytotoxicity. Ubiquitous distributions of CD46 molecules among normal tissues however need an additional system to secure tumor specificity in AdF35-mediated transduction. The transcriptional regulatory regions of the *MK* and the *Sur* can activate an exogenous gene with tumor selectivity and we previously demonstrated preferential cytotoxicity of replication-competent Ad5 in tumors when the *E1A* gene was activated by MK or Sur promoter region [11–14]. The promoter regions were active in esophageal carcinoma (Additional file 5: Table S3), but the transcriptional activity may not be directly linked with the Ad-mediated cytotoxicity since multiple factors such as sensitivity to cell death are involved in the cytotoxicity. Our previous pathological analyses also showed massive necrosis and pyknotic changes in Ad-injected tumors but the injected non-tumorous regions remained unchanged [14]. On the other hand, regulation of pRb phosphorylation was complex in Ad-infected cells. YES-2 cells showed hyper-phosphorylated pRb, which could favor viral replications through enhanced cell cycle progression. In contrast, hypo-phosphorylation of pRb in T.Tn cells rather suggested cell cycle arrest prior to cell death. These phosphorylation statuses however did not accord with the actual cell cycle data in Additional file 2: Table S1 and further investigations are required as for pRb regulation in Ad replications and subsequent cell death.

Combinatory effects of replication-competent Ad and forced expression of p53 can be examined either with dual transduction using 2 kinds of Ad vectors or with a single vector system in which the *p53* gene was integrated into the replication-competent Ad vector. Different groups using a dual transduction system demonstrated that combinatory cytotoxicity was achieved with replication-incompetent Ad5/p53 and either with wild-type Ad5 [19] or replication-competent Ad5 that were activated by telomerase reverse transcriptase (TERT) promoter [20]. These studies however used the same type of Ad5 vectors in dual transduction and did not consider possibility of decreased infectivity. Moreover, previous studies with a replication-competent Ad5 vector which had the wild-type *p53* gene and was activated by Sur [21] or TERT promoter [22, 23] demonstrated greater cytotoxicity than Ad5/p53 or the promoter-activated Ad5 alone. These previous studies however did not analyze the combinatory effects in contrast to the present study that demonstrated the synergism. The p53-bearing single vector system in the previous study also showed that production of viral progenies was uninhibited by the expressed p53 and suggested that infection-induced down-regulated p21 and Mdm-2 were associated with Ad-mediated apoptosis and autophagy [22, 23]. In contrast, the present study demonstrated that production of AdF35/MK or AdF35/Sur progenies was rather inhibited by co-transduced p53, which was probably linked with p53-mediated decrease of cell growth and acceleration of cell death.

Cell death mechanisms mediated by replication-competent Ad are complex. The present study showed that the cytotoxicity was not attributable to autophagy but to apoptosis although activation of the intrinsic and/or the extrinsic apoptotic pathways was dependent on cells types tested. Yamasaki *et al*. showed that OBP-702, p53-expressing and TERT promoter-activated replication-competent Ad induced apoptosis in lung and esophageal carcinoma cells [22], but Hasei *et al*. demonstrated with the same OBP-702 killed osteosarcoma cells through autophagy [23]. These previous studies thus indicated differential cell death mechanisms even with the same Ad construct. Cell death due to Ad replication was influenced by balance between pro-apoptotic and anti-apoptotic signaling, and was partly linked with Ad-derived E1B-19 kDa molecules [24]. Recent studies also demonstrated that an elevated caspase activity in the extrinsic pathway contributed to Ad-induced autophagy-mediated cell death [25]. These reports together with the present data suggest that Ad replication-mediated cell death is controlled by multiple factors including genetic differences of the infected cells, which is resulted in varied levels of susceptibility to apoptosis and autophagy. Our preliminary study with an inhibitor for necrosis suggested that necrosis was not involved in Ad replication-induced cell death, but further investigation is required to clarify the involvement of necrotic cell death.

We demonstrated that phosphorylation of Ad-induced p53 was augmented by co-infected replication-competent AdF35 although the AdF35 themselves did not induce phosphorylation of endogenous p53. A possible upstream pathway to phosphorylate p53 remains uncharacterized, but Ad proteins synthesized during the replications such as Ad E1B-55 kDa and E4-ORF3 may regulate p53 expression at various levels even in an epigenetic manner [26]. Regulation of the p53 pathways after Ad infection is controlled even by a host defense system such as interferons [27]. A biomarker to predict the efficacy of Ad-mediated cytotoxicity to target tumors as well as detection of Ad ability to penetrate into tumors is preferable in the case of its clinical application. Ad infectivity can be one of the factors involved in the cytotoxicity but is not completely dependent on the receptor expression levels because an auxiliary receptor play a role in Ad infection and the threshold level necessary for the infection can be different among the target cells [18]. It is crucial to examine a possible correlation between the infectivity and anti-tumor effects with clinical specimens. Our different results showed that an Ad5 injection into subcutaneous tumors did not affect the subsequent gain of mice body weight but only elevated transaminases with a mild degree, suggesting the feasibility of a clinical trial.

We noticed that replication-competent AdF35 induced hyperploid populations which preceded increase of sub-G1 fractions. Cherubini *et al*. showed that replication-competent Ad with deletion of E1B-55 kDa molecules induced hyperploidy populations in normal cells, and suggested a possible linkage between the hyperploidy and increase expression of mitotic arrest-deficient 2 molecules [28], a major component of the M-phase checkpoint [29]. The study also indicated that wild-type Ad5 did not induce the hyperploidy and subsequently ruled out a possibility that the hyperploidy was caused by amplified viral DNA contents [28]. Our previous study also found the E1B-55 kDa-defective Ad induced similar hyperploidy in mesothelioma cells without any changes at the chromosome levels [30]. Our preliminary data also showed that the wild-type Ad minimally induced hyperploidy but rapidly increased sub-G1 fractions in esophageal carcinoma (data not shown). These data suggest that Ad replication processes per se are not directly associated with hyperploidy, but that differential cell death mechanisms, which are influenced by a number of genetic difference in infected cells, may also play a role in the aberrant cell cycle progression. Investigations on genesis of the hyperploidy require genetically modified cells to standardize the genetic backgrounds.

## Conclusions

Esophageal carcinoma can be an ideal target for gene therapy. Several studies demonstrated the clinical feasibility [31, 32] and our previous clinical study with Ad5/p53 showed that direct administration of Ad produced none of the major adverse effects [33]. We demonstrated that combination of replication-incompetent Ad5/p53 and replication-competent AdF35 achieved synergistic cytotoxicity through activated the p53 pathways although replication of AdF35 itself was suppressed. The present study suggests that administration of the dual vectors at an optimized ratio, perhaps being personalized for the p53 expression in an individual tumor, can broaden therapeutic options for esophageal carcinoma, which includes further combination with chemotherapy and radiotherapy.

## Additional files

Additional file 1: Figure S1. Enhanced cytotoxicity of AdF35/Sur. Viability of esophageal carcinoma cells that were treated with various dose of Ad5/Sur, AdF35/Sur, Ad5/LacZ or AdF35/LacZ was examined with the WST assay. The relative viability was calculated based on the absorbance without any treatments. SE bars are shown (*n* = 3). ^*^*P* < 0.01; comparing between Ad5/Sur- or AdF35/Sur-infected cells and Ad5/LacZ- or AdF35/LacZ-infected cells.
Additional file 2: Table S1. Cell cycle distribution after AdF35 infections.
Additional file 3: Figure S2. Anti-tumor effects *in vivo* produced by AdF35/MK or AdF35/Sur. Nude nice that were subcutaneously injected with YES-2 cells (1x10^6^) were treated on day 7, 10, 13 and 16 with intra-tumoral injection of AdF35/LacZ, AdF35/MK or AdF35/Sur (2.5x10^8^ pfu/mouse), or with phosphate buffered-saline (PBS) as a control. The tumor sizes were measured every 4 days. Means and SE bars are shown. ^*^*P* < 0.01.
Additional file 4: Table S2. Cell cycle distribution after combinatory Ad infections.
Additional file 5: Table S3. Transcriptional activity of MK and Sur regulatory regions.

## Abbreviations

Ab: Antibody
Ad: Adenoviruses
Ad5: Type 5 adenoviruses
Ad35: Type 35 adenoviruses
AdF35: Type 5 adenoviruses with type 35-derived fiber-knob
CAR: Coxsackie adenovirus receptor
CI: Combination index
DR5: Death receptor 5
Fa: Fractions affected
FADD: Fas-associated protein with death domain
GAPDH: Glyceraldehyde-3-phosphate dehydrogenase
GFP: Green fluorescent protein
HEK: Human embryonic kidney
MK: Midkine
MOI: Multiplicity of infection
PARP: Poly (ADP-ribose) polymerase
pfu: Plaque-forming unit
Sur: Surviving
t-Bid: Truncated Bid
TCID_50_: Median tissue culture infectious dose
TERT: Telomerase reverse transcriptase
vp: Virus particles
