# Supplementary figures and images for: Cytotoxic effects of replication-competent adenoviruses on human esophageal carcinoma are enhanced by forced p53 expression
Source: BMC Cancer. 2015 Jun 10;15:464. doi: 10.1186/s12885-015-1482-8 (PMC4460641; doi:10.1186/s12885-015-1482-8)

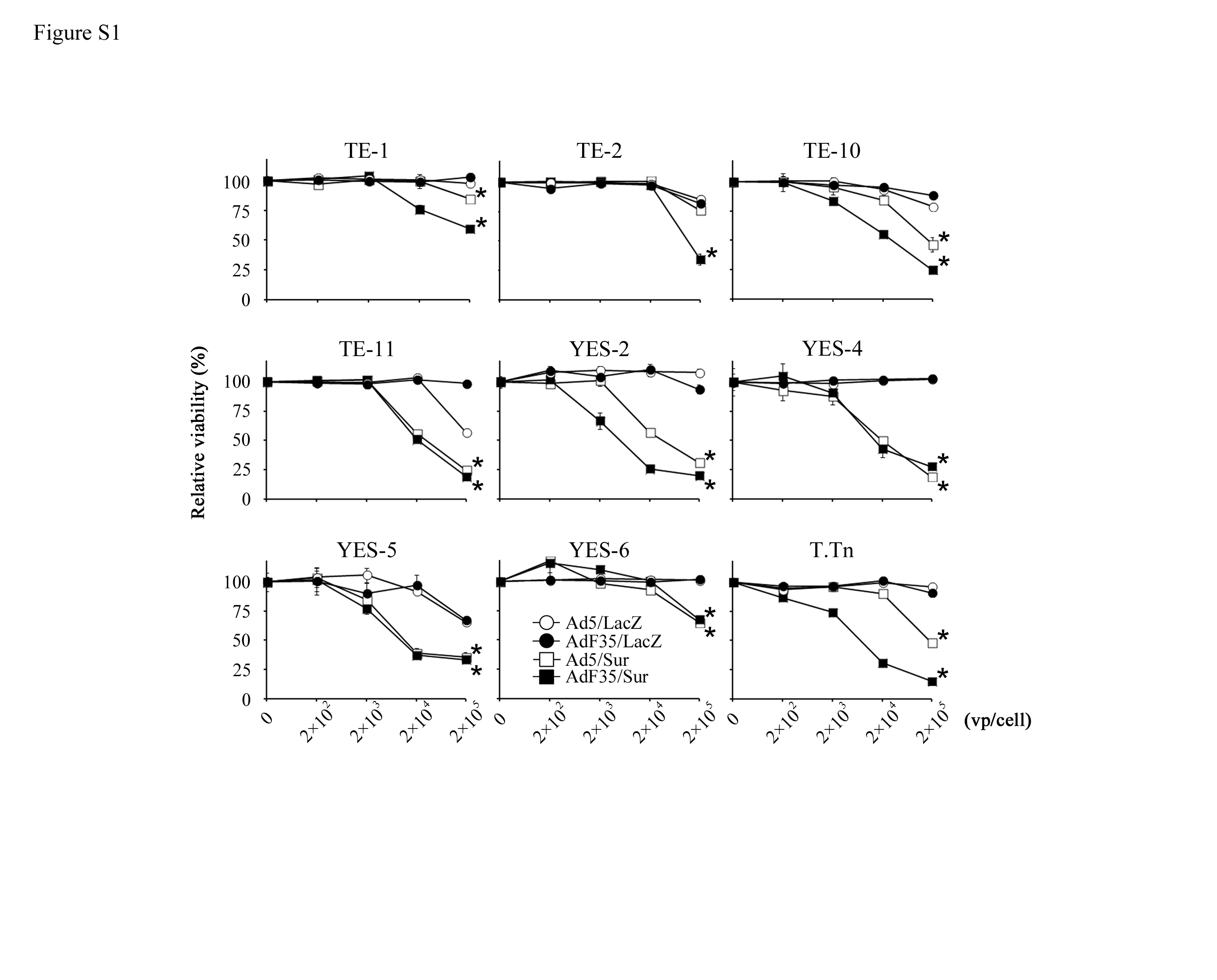

Supplement: Additional file 1: Figure S1. — Enhanced cytotoxicity of AdF35/Sur. Viability of esophageal carcinoma cells that were treated with various dose of Ad5/Sur, AdF35/Sur, Ad5/LacZ or AdF35/LacZ was examined with the WST assay. The relative viability was calculated based on the absorbance without any treatments. SE bars are shown (n = 3). *P < 0.01; comparing between Ad5/Sur- or AdF35/Sur-infected cells and Ad5/LacZ- or AdF35/LacZ-infected cells. [file 12885_2015_1482_MOESM1_ESM.tiff]

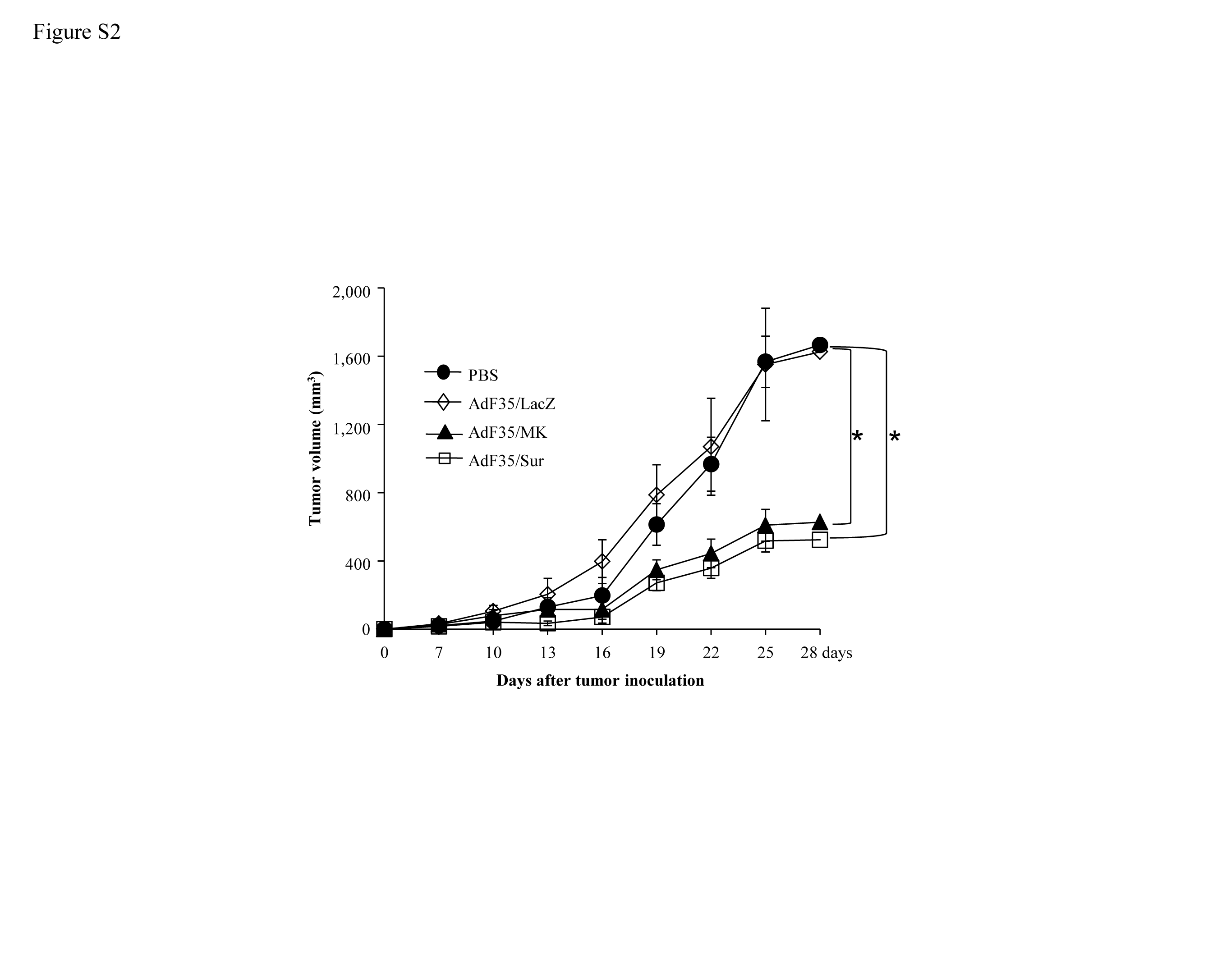

Supplement: Additional file 3: Figure S2. — Anti-tumor effects in vivo produced by AdF35/MK or AdF35/Sur. Nude nice that were subcutaneously injected with YES-2 cells (1x10^6^) were treated on day 7, 10, 13 and 16 with intra-tumoral injection of AdF35/LacZ, AdF35/MK or AdF35/Sur (2.5x10^8^ pfu/mouse), or with phosphate buffered-saline (PBS) as a control. The tumor sizes were measured every 4 days. Means and SE bars are shown. *P < 0.01. [file 12885_2015_1482_MOESM3_ESM.tiff]
