# Supplementary material for: Cytotoxic effects of replication-competent adenoviruses on human esophageal carcinoma are enhanced by forced p53 expression
Source: BMC Cancer. 2015 Jun 10;15:464. doi: 10.1186/s12885-015-1482-8 (PMC4460641; doi:10.1186/s12885-015-1482-8)
Supplement: Additional file 2: Table S1. — Cell cycle distribution after AdF35 infections. [file 12885_2015_1482_MOESM2_ESM.doc]

**Table S1. Cell cycle distribution after AdF35 infections**

| Cells | Treatment | Time (day) | Cell cycle distribution (%) | | | | |
| --- | --- | --- | --- | --- | --- | --- | --- |
|  |  |  | Sub-G1 | G0/G1 | S | G2/M | >4N |
| T.Tn | (-) | 3 | 1.49±0.08 | 70.00±0.17 | 16.33±0.16 | 11.16±0.08 | 1.45±0.06 |
|  | Ad5F35/LacZ | 3 | 1.49±0.12 | 65.42±0.59 | 19.27±0.38 | 12.17±0.38 | 2.13±0.05 |
|  | AdF35/MK | 3 | 5.68±0.09^a^ | 46.84±0.26 | 13.99±0.06 | 12.95±0.06 | 20.95±0.34^a^ |
|  | AdF35/Sur | 3 | 6.88±0.10^a^ | 60.01±0.05 | 13.80±0.26 | 10.61±0.22 | 9.02±0.16^a^ |
|  | (-) | 4 | 1.70±0.07 | 51.05±0.25 | 18.83±0.15 | 26.10±0.14 | 2.81±0.16 |
|  | Ad5F35/LacZ | 4 | 2.79±0.06 | 66.30±0.33 | 14.39±0.37 | 15.18±0.20 | 1.81±0.06 |
|  | AdF35/MK | 4 | 15.82±0.16^a^ | 37.22±0.55 | 13.95±0.11 | 11.44±0.20 | 22.09±0.11^a^ |
|  | AdF35/Sur | 4 | 12.78±0.41^a^ | 53.32±0.50 | 13.35±0.29 | 10.40±0.17 | 10.67±0.07^a^ |
|  | (-) | 5 | 1.86±0.15 | 71.44±0.17 | 12.17±0.12 | 12.61±0.17 | 2.20±0.09 |
|  | Ad5F35/LacZ | 5 | 1.34±0.10 | 72.30±0.11 | 14.27±0.03 | 10.93±0.14 | 1.49±0.02 |
|  | AdF35/MK | 5 | 34.64±0.08^a^ | 30.02±0.21 | 15.80±0.22 | 7.64±0.27 | 12.72±0.21^a^ |
|  | AdF35/Sur | 5 | 32.80±0.27^a^ | 42.01±0.06 | 14.30±0.17 | 6.37±0.15 | 5.07±0.05^a^ |
| YES-2 | (-) | 2 | 2.44±0.01 | 46.67±0.62 | 23.67±0.49 | 24.88±0.15 | 3.00±0.08 |
|  | Ad5F35/LacZ | 2 | 3.01±0.06 | 44.78±0.20 | 23.42±0.15 | 25.70±0.25 | 3.75±0.12 |
|  | AdF35/MK | 2 | 5.28±0.05^a^ | 5.89±0.10 | 7.11±0.15 | 12.13±0.18 | 69.95±0.27^a^ |
|  | AdF35/Sur | 2 | 5.51±0.16^a^ | 11.17±0.12 | 9.31±0.07 | 14.07±0.08 | 60.33±0.20^a^ |
|  | (-) | 3 | 3.54±0.11 | 51.39±0.44 | 21.19±0.35 | 21.55±0.33 | 2.97±0.03 |
|  | Ad5F35/LacZ | 3 | 4.60±0.02 | 52.04±0.47 | 20.81±0.41 | 18.89±0.28 | 4.36±0.37 |
|  | AdF35/MK | 3 | 16.92±0.59^a^ | 2.20±0.10 | 4.45±0.14 | 4.13±0.21 | 72.40±0.29^a^ |
|  | AdF35/Sur | 3 | 28.32±0.08^a^ | 2.22±0.04 | 2.13±0.06 | 3.41±0.15 | 64.12±0.25^a^ |
|  | (-) | 4 | 2.45±0.05 | 57.75±0.13 | 22.19±0.32 | 14.87±0.39 | 3.32±0.12 |
|  | Ad5F35/LacZ | 4 | 2.65±0.14 | 58.02±0.75 | 21.64±0.25 | 15.01±0.58 | 3.26±0.13 |
|  | AdF35/MK | 4 | 32.18±0.23^a^ | 2.34±0.02 | 1.93±0.05 | 1.44±0.10 | 62.23±0.28^a^ |
|  | AdF35/Sur | 4 | 53.86±1.31^a^ | 8.42±1.42 | 10.35±0.42 | 7.58±052 | 20.22±1.06^a^ |

Cells were infected with AdF35/MK, AdF35/Sur or AdF35/LacZ (1×10^4^ vp/cell) or uninfected, and then were cultured for the indicated time. Cells cycle profiles were analyzed with flow cytometry. Mean percentages with SEs are shown (n=3).

^a^ P<0. 01; comparing between AdF35/MK- or AdF35/Sur-infected cells and uninfected or AdF35/LacZ-infected cells.
