# Supplementary material for: Cytotoxic effects of replication-competent adenoviruses on human esophageal carcinoma are enhanced by forced p53 expression
Source: BMC Cancer. 2015 Jun 10;15:464. doi: 10.1186/s12885-015-1482-8 (PMC4460641; doi:10.1186/s12885-015-1482-8)
Supplement: Additional file 4: Table S2. — Cell cycle distribution after combinatory Ad infections. [file 12885_2015_1482_MOESM4_ESM.doc]

**Table S2. Cell cycle distribution after combinatory Ad infections**

| Treatment | Time (day) | Cell cycle distribution (%) | | | | |
| --- | --- | --- | --- | --- | --- | --- |
|  |  | Sub-G1 | G0/G1 | S | G2/M | >4N |
| (-) | 2 | 2.08±0.31 | 53.33±0.54 | 28.07±1.23 | 14.39±1.02 | 2.52±0.15 |
| Ad5/LacZ | 2 | 1.96±0.17 | 44.86±0.94 | 31.38±0.81 | 18.81±1.21 | 3.42±0.60 |
| Ad5/p53 | 2 | 7.53±0.16 | 58.62±0.63 | 16.00±0.23 | 17.21±0.28 | 1.05±0.16 |
| AdF35/MK | 2 | 4.18±0.05 | 28.05±0.47 | 21.37±0.45 | 22.40±0.34 | 24.47±0.16 |
| AdF35/MK+Ad5/LacZ | 2 | 4.12±0.06 | 29.28±0.12 | 22.03±0.23 | 19.97±0.30 | 25.04±0.35 |
| AdF35/MK+Ad5/p53 | 2 | 28.83±0.37^a^ | 31.77±0.29 | 18.93±0.08 | 16.88±0.36 | 4.03±0.14^b^ |
| (-) | 3 | 2.06±0.16 | 54.45±0.19 | 28.53±0.13 | 13.76±0.21 | 1.62±0.26 |
| Ad5/LacZ | 3 | 1.22±0.04 | 46.56±0.28 | 35.30±0.09 | 14.82±0.42 | 2.44±0.38 |
| Ad5/p53 | 3 | 3.09±0.19 | 55.83±0.25 | 19.62±0.36 | 20.75±0.36 | 1.13±0.04 |
| AdF35/MK | 3 | 8.97±0.08 | 25.02±0.05 | 26.06±0.28 | 19.28±0.34 | 21.30±0.21 |
| AdF35/MK+Ad5/LacZ | 3 | 5.65±0.52 | 22.14±0.52 | 23.38±0.48 | 21.13±0.36 | 28.27±1.06 |
| AdF35/MK+Ad5/p53 | 3 | 73.71±0.41^a^ | 12.41±0.48 | 9.14±0.08 | 2.94±0.18 | 2.16±0.05^b^ |

YES-2 cells were uninfected or infected with AdF35/MK (1.2×10^3^ vp/cell), Ad5/p53, Ad5/LacZ (6.5×10^3^ vp/cell) or in combination and were cultured for the indicated time. Cell cycle profiles were analyzed with flow cytometry. Mean percentages with SEs are shown (n=3).

^a^ P<0. 01; comparing between AdF35/MK+Ad5/p53-infected cells and AdF35/MK-, Ad5/p53- or AdF35/MK+Ad5/LacZ-infected cells regarding sub-G1 fraction.

^b^ P<0. 01; comparing between AdF35/MK+Ad5/p53-infected cells and AdF35/MK- or AdF35/MK+Ad5/LacZ-infected cells regarding hyperploidy fraction.
