# Supplementary material for: Cytotoxic effects of replication-competent adenoviruses on human esophageal carcinoma are enhanced by forced p53 expression
Source: BMC Cancer. 2015 Jun 10;15:464. doi: 10.1186/s12885-015-1482-8 (PMC4460641; doi:10.1186/s12885-015-1482-8)
Supplement: Additional file 5: Table S3. — Transcriptional activity of MK and Sur regulatory regions. [file 12885_2015_1482_MOESM5_ESM.docx]

**Table S3 Transcriptional activity of MK and Sur regulatory regions**

Cells Luciferase activity (% average+SE)

MK Sur

TE-1 400+99 162+14

TE-2 375+8 610+137

TE-10 339+27 906+35

TE-11 702+30 676+53

YES-2 314+20 178+16

YES-4 187+3 309+13

YES-5 744+47 394+11

YES-6 540+7 311+10

T.Tn 198+32 240+23

# The genomic DNA fragments of the *MK* or the Sur gene was cloned into the pGL2-basic vector (Promega, Madison, WI) that contained the *firefly luciferase* gene. The plasmid DNA was transfected into esophageal carcinoma cells with a lipofectin reagent (Life Technologies, Gaithersburg, MD). The luciferase activities from cell lysate were measured with a dual-luciferase reporter assay (Promega) and standardized with the transfection efficacy with a control vector bearing *renilla luciferase* gene. The value was expressed as a percent activity of the SV40 T antigen promoter (pGL-2 control, Promega).
